# Hip displacement in relation to age and gross motor function in children with cerebral palsy

**DOI:** 10.1007/s11832-014-0570-7

**Published:** 2014-03-05

**Authors:** Per Larnert, Olof Risto, Gunnar Hägglund, Philippe Wagner

**Affiliations:** 1 Division for Clinical and Experimental Medicine, Department of Orthopaedics, Linköping University, 585 81 Linköping, Sweden; 2 Swedish National Competence Centre for Musculoskeletal Disorders, Lund University Hospital, Lund, Sweden; 3 Department of Orthopaedics, Lund University Hospital, Lund, Sweden

**Keywords:** Cerebral palsy, CP, Hip displacement, Hip dislocation, Hip surveillance

## Abstract

**Purpose:**

Hip dislocation in cerebral palsy (CP) is a serious complication. By radiographic screening and prophylactic surgery of children at risk most dislocations can be prevented. CPUP, the Swedish CP registry and follow-up program, includes annual radiographic examinations of children at Gross Motor Function Classification System (GMFCS) levels III–V. Data from CPUP were analysed to assess the risk of hip displacement in relation to GMFCS levels and age.

**Methods:**

All children at GMFCS levels III–V (*N* = 353) whose first radiographic screening occurred before 3 years of age were followed between the ages 2–7 years. Migration percentages (MPs) were recorded annually (1,664 pelvic radiographs) and analysed using discrete time survival analysis.

**Results:**

The risk of hip displacement between 2 years and 7 years of age was significantly (*p* < 0.05) higher for children at GMFCS level V during the entire study period. The risk was highest at 2–3 years of age and decreased significantly (*p* < 0.001) with each year of age (OR = 0.71, 95 % CI 0.60–0.83). The cumulative risk at age 7 years for those at GMFCS V for MP ≥ 40 % was 47 % (95 % CI 37–58). The corresponding risk at GMFCS IV was 24 % (16–34) and at GMFCS III 23 % (12–42).

**Conclusions:**

Children at GMFCS V have a significantly higher risk of hip displacement compared with children at GMFCS III–IV. The risk is highest at 2–3 years of age. The results support a surveillance program including radiographic hip examinations as soon as the diagnosis of severe CP is suspected.

## Introduction

Hip dislocation in cerebral palsy (CP) often causes severe suffering, including pain [1–3]. Reduced range of hip motion with associated sitting, standing and walking problems [4, 5] are common. There is also an association between hip dislocation and pelvic obliquity, windswept deformity, and scoliosis [6].

Hip displacement is reported to affect 7 % of walking and 60 % of total body involved children [7]. Increased spasticity and shortening of the psoas and hip adductor muscles in relation to the hip abductors and extensors is regarded as the primary pathology [8].

**Fig. 1:**
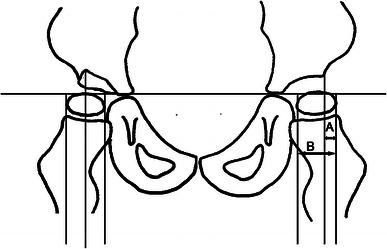
Measurement of Migration Percentage (MP). MP = A/B × 100. On the right hip with a “Gothic arch” formation of the lateral margin, the midpoint of the arch is used as reference point

The degree of hip displacement in CP is generally measured by Reimers’ migration percentage (MP) [9]. The MP describes the percentage of the ossified femoral head positioned lateral to the acetabular margin on an anteroposterior pelvic radiograph (Fig. 1). Gross motor function in CP is usually assessed according to the Gross Motor Function Classification System (GMFCS) [10], a five-level ordinal scale in which level I corresponds to the highest and level V to the lowest level of function.

In 1994, a hip surveillance program for children with CP was initiated in southern Sweden and the results from the first ten years of the program were presented in 2005 [11]. Those findings now provide the underlying basis for intensified observation in the MP 33–40 % interval and surgical treatment in hips with MP >40 %. In 2005, the surveillance program also became a Swedish national quality registry [12]. CPUP, and other surveillance programs for early detection have been found to reduce the incidence of hip displacement in CP [13].

The risk of progression of hip displacement is increased with more severe motor impairment [14–16], and also increases with a MP above certain threshold values [17]. Prophylactic soft tissue surgery seems to be most effective before 4 years of age [18–20], indicating that age probably is a key factor in preventing hip displacement.

The aim of this study was to analyse the risk of hip displacement in relation to age and gross motor function in a total population of children with CP at GMFCS levels III–V.

## Materials and methods

The CPUP registry includes all children with CP born 1990 and onwards residing in the counties of Skåne and Blekinge (population 1.4 million) and in all of Sweden (population 9.4 million) born 2001 and onwards. The CPUP surveillance program includes annual radiographic hip examinations of children at GMFCS levels III, IV and V starting at the first suspicion of a possible CP diagnosis. The degree of hip displacement is measured by MP (Fig. 1) [9].

All children in the CPUP registry at GMFCS levels III–V who had their first radiographic hip examination performed before 3 years of age were included in the present study. Children having this first radiograph before the end of 2010 were included.

The risk of either hip exceeding a MP of 33 % or 40 %, respectively, was studied between the ages of 2–7 years. Hips were assessed until the date of any hip surgery.

Analysis of change in risk was performed using discrete time survival analysis. GMFCS levels were entered as the indicator variable and age (continuous) was included as a covariate into a logistic regression model to calculate odds ratios (OR) with corresponding confidence intervals (CI) to test observed difference in risk. The *p* values <0.05 were considered significant. The model fit was measured using the Pearson goodness-of-fit test in STATA 12 [21]. Analogous analyses were performed for both the risk of exceeding 33 % and exceeding or obtaining exactly 40 %. The reason for including the 40 % level in the event definition was because of the low number of children that exceeded 40 %.

The life table method applied in 1 year intervals was used to compute the discrete hazard (incidence) and failure time distribution (cumulative risk) corresponding to the MP levels 33 % and 40 %. The hazard may be interpreted as the risk of the worst hip exceeding an MP of 33 % (or 40 %) at an examination at a particular age given that the child has not experienced the event, or has had surgery, before that age. The failure time distribution shows the cumulative risk of having exceeded 33 % or 40 % at a given age, i.e., the expected proportion to have experienced the event at a given age.

The study was approved by the Medical Research Ethics Committee at Lund University (LU-443-99).

## Results

The results are based on 1,664 radiographic examinations of 353 children. In the total study population, 17 % (95 % CI 14–23) of the children had MP > 33 % and 11 % (95 % CI 8–15) had MP > 40 % at 2–3 years of age (up to the third birthday). The annual incidence decreased with age and was zero for MP > 33 % and 0.05 % for MP > 40 % at 7–8 years of age. At 7 years of age, the cumulative risk of having developed a hip with MP > 33 % was 44 % (95 % CI 38–51) and the risk related to MP > 40 % was 33 % (95 % CI 2740).

The annual incidence of hip displacement was significantly (*p* < 0.05) higher in children at GMFCS level V compared to those at GMFCS level III and IV. At 2–3 years of age, 23 % of children in GMFCS V had a MP > 33 % and 17 % had a MP > 40 % (Tables 1, 2). The corresponding figures for children in GMFCS IV were 13 % and 7 %, respectively, and for children in GMFCS III, 16 % and 5 %.

**Table 1:** Annual incidence for hip displacement >33 % in relation to GMFCS level

Age (years)	GMFCS III		GMFCS IV		GMFCS V	
	Incidence	95 % CI	Incidence	95 % CI	Incidence	95 % CI
2–3	0.16	0.08–0.27	0.13	0.07–0.20	0.23	0.15–0.32
3–4	0.04	0.00–0.11	0.11	0.05–0.18	0.23	0.13–0.34
4–5	0.09	0.02–0.21	0.10	0.04–0.19	0.20	0.10–0.34
5–6	0.04	0.00–0.16	0.04	0.00–0.10	0.08	0.01–0.21
6–7	0		0		0.24	0.06–0.52
7–8	0		0		0

**Table 2:** Annual incidence for hip displacement >40 % in relation to GMFCS level

Age (years)	GMFCS III		GMFCS IV		GMFCS V	
	Incidence	95 % CI	Incidence	95 % CI	Incidence	95 % CI
2–3	0.05	0.01–0.12	0.07	0.03–0.13	0.17	0.11–0.25
3–4	0.09	0.03–0.18	0.08	0.04–0.15	0.16	0.09–0.25
4–5	0		0.04	0.01–0.09	0.13	0.06–0.23
5–6	0		0.02	0.00–0.06	0.12	0.03–0.27
6–7	0		0.02	0.00–0.08	0	
7–8	0.11	0.01–0.31	0.03	0.00–0.12	0	

**Fig. 2:**
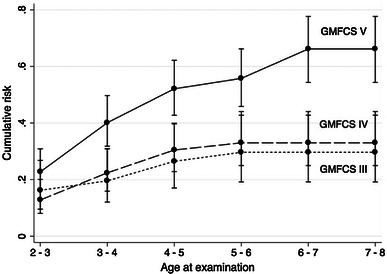
Cumulative risk of hip displacement MP > 33 %

**Fig. 3:**
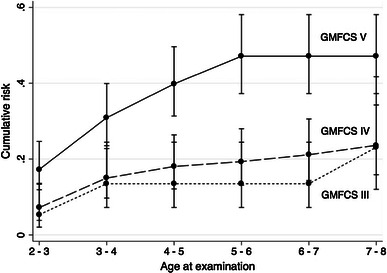
Cumulative risk of hip displacement MP ≥ 40 %

The cumulative risk for hip displacement above 33 % at 7–8 years of age for children at GMFCS level V was 66 % (95 % CI 54–78). For displacement >40 % the cumulative risk was 47 % (95 % 37–58) (Figs. 2, 3). The corresponding figures for children in GMFCS IV were 33 % and 24 % and for children in GMFCS III, 30 % and 23 %.

The OR of hip displacement up to 8 years of age was significantly increased for children in GMFCS level V compared to level III and IV. For children with MP > 33 % the OR was 2.6 (CI 1.5–4.5, *p* = 0.001) and 2.3 (CI 1.5–3.6, *p* < 0.001), respectively. The difference between GMFCS III and IV was not significant. The results for displacement MP > 40 % was similar (Tables 3, 4).

**Table 3:** Odds Ratio (OR) for exceeding MP > 33 % in relation to GMFCS-levels

GMFCS	Odds Ratio	Standard-error	*Z*	*P*	95 % CI
4 vs 3	1.10	0.33	0.30	0.762	0.61–1.99
5 vs 3	2.56	0.73	3.29	0.001	1.46–4.49
5 vs 4	2.34	0.53	3.77	<0.001	1.50–3.64
Age group	0.71	0.59	−4.14	<0.001	0.60–0.83

**Table 4:** Odds Ratio (OR) for exceeding MP > 40 % in relation to GMFCS-levels

GMFCS	Odds Ratio	Standard-error	*Z*	*P*	95 % CI
4 vs 3	1.20	0.45	0.49	0.625	0.57–2.50
5 vs 3	3.21	1.11	3.38	0.001	1.63–6.32
5 vs 4	2.67	0.70	3.77	<0.001	1.60–4.46
Age group	0.75	0.69	−3.14	0.002	0.63–0.90

## Discussion

This study is based on data from CPUP—the Swedish national quality register and follow-up program for CP were 95 % of all children with CP participate [12]. The registry data are collected through the CPUP follow-up programme.

Children at GMFCS V had a 2.5–3 times higher risk of developing hip displacement compared with children at GMFCS III–IV. Children at GMFCS V had the highest annual incidence already at age 2–3 years and at 7–8 years of age 66 % of children at GMFCS V had developed displacement >33 %.

The annual incidence of displacement decreased by age and was very low in the 7–8 years age-group. Later displacement is unlikely, as has been shown by others [8, 14], in children with normal hips by this age, given that they have not previously needed preventive surgery.

The use of MP as the primary variable for measurement of hip displacement in CP is well documented [11, 13, 15, 16, 22]. The GMFCS level is stable over time [23–25], especially for children in GMFCS I and V, and can be assessed starting at 2 years of age [24, 25]. Radiographic signs of hip displacement can often be detected already at this age [14]. We chose to use the GMFCS to assess the relationship between hip displacement and severity of CP rather than CP-subtype which sometimes is difficult to classify [26], at least before 4 years of age [14].

The threshold values used in this study are based on findings from an earlier study [17]. One-third of hips with MP 33–39 % improved below the threshold MP 33 % without surgical treatment, but only one of ten hips with MP ≥ 40 % improved below that threshold without surgery.

Several factors that are likely to influence the natural history of hip displacement have not been addressed in our study. The use of postural management equipment has been reported to reduce the risk of displacement [27]. A preventative effect of weight bearing in abduction and extension has also been suggested [28]. This kind of treatment is part of the standard treatment protocol for children with CP in Sweden. Another possible confounding factor is treatment with Botulinum toxin A, even though the effect on the hip displacement process in CP has been questioned [29].

Faraj and colleagues found the inter-observer error for the MP for less experienced observers to be a median difference of as low as 2.8 %, but the upper 95 % confidence interval was up to 22.4 % [30]. According to Parrot et al. [31], an experienced observer is expected to measure MP within 5.8 % of the true value and they found an inter-observer worst agreement of 6.5 %. Measurement of MP within CPUP is done by consultant orthopaedic surgeons with a particular interest in neuropaediatric conditions or by radiologists at an equal level of experience, in all counties throughout Sweden. A majority use measurement tools integrated in the digital radiology systems, PACS (Picture Archiving and Communication System), which has been shown to be at least as reliable as manual measurements [32].

There could be a selection bias of our material as children with the most severe motor impairment might get an earlier radiographic examination. Given that GMFCS allows reliable grading of motor impairment already at 2 years of age, we believe it unlikely that more than a small number of children with GMFCS III–V would not have received a CP diagnosis by then. Another possible selection bias is children who had surgery in the MP 33–39 % interval. The proportion of patients with displacement above MP 40 % would then be underestimated. Furthermore, this is a very rare event in clinical practice in Sweden.

Terjesen examined radiographs of 76 children with bilateral spastic CP up to the first surgical intervention [16]. The average annual increase in MP for children with tetraplegic CP was 13 % before 5 years of age and 7 % after age 5. No such difference could be shown for children with diplegic CP. Scrutton et al., measured multiple radiographic variables at 18, 24, 30, 48, and 60 months of age in 346 children with bilateral CP [22]. Besides concluding that MP, measured correctly, is the best guide to hip surveillance and the need for treatment, they showed that MP was significantly greater in their cohort than in normally developing children already at 18 months of age. Our results confirm these authors’ findings that the tendency for displacement is more pronounced at earlier ages in children with severe motor impairment.

Our results confirm other findings of the markedly increased risk of displacement in children with severe motor impairment [14–16]. In addition we found a statistically significant difference in risk of hip displacement in children at GMFCS V compared to GMFCS III and IV, but no difference between levels III and IV. One explanation could be that children at GMFCS III start walking after the age-period of our significant findings. Almost all children in GMFCS V have spastic tetraplegic or dyskinetic subtypes [33], while a majority of children in GMFCS III and IV have spastic diplegic CP, which is another possible explanation of the difference in risk between these levels. Soo et al. [15] also reported an increased risk of displacement above MP 30 % related to GMFCS-levels, and related to CP-subtype with the highest risk in children with spastic quadriplegia (83 %) and dystonic subtype (40 %), compared with spastic diplegia (19 %).

In conclusion, children at GMFCS V had a significantly higher risk of hip displacement, and they also displaced at an earlier age than children at GMFCS III or IV. To be able to prevent hip displacement in those at highest risk in a surveillance program it is of outmost importance that children with suspected or confirmed CP have a radiographic hip examination undertaken as soon as possible.
